# Analysis of Pressure Fluctuation Characteristics of Central Swirl Combustors Based on Empirical Mode Decomposition

**DOI:** 10.3390/s22155615

**Published:** 2022-07-27

**Authors:** Xuhuai Wang, Xiang Zhang, Chen Yang, Hao Li, Yong Liu

**Affiliations:** Key Laboratory of Aero-Engine Thermal Environment and Structure, Ministry of Industry and Information Technology, College of Energy and Power Engineering, Nanjing University of Aeronautics and Astronautics, Nanjing 210016, China; limusanjin@nuaa.edu.cn (X.W.); zhangxiang0321@nuaa.edu.cn (X.Z.); ycyc66666@163.com (C.Y.); leelihao@126.com (H.L.)

**Keywords:** swirl combustor, dynamic pressure sensors, photoelectric sensor, empirical mode decomposition, intrinsic mode function

## Abstract

In order to study the characteristics of pressure fluctuation during unstable combustion, experimental studies had been conducted on the mechanism model of the swirl combustor and the industrial swirl combustor. The signal of dynamic pressure, heat release rate, and the high-speed flame image in the two combustors were synchronously collected by using dynamic pressure sensors, a photoelectric sensor, and a high-speed camera under normal temperature and pressure. After empirical mode decomposition of the dynamic pressure signal, several intrinsic mode functions were obtained. It was found that the pressure pulsation energy is concentrated in the first three order intrinsic mode function. Through fast Fourier transform spectrum calculation, it was found that the first three order intrinsic mode function pulsation can characterize the changes of heat release rate and air flow pulsation under cold state and flame instability. It showed that the decomposition of the dynamic pressure in the combustor by this method can obtain the main physical processes in its connotation, and provide data processing methods for the induction mechanism of oscillating combustion and combustion diagnosis in an industrial combustor test.

## 1. Introduction

With increasing environmental problems, the requirements for polluting emissions from modern aeronautical engines are becoming stricter. In order to reduce pollutant emissions, one of the most commonly used combustion methods is central staged swirl lean fuel combustion, but lean fuel combustion will induce unstable combustion and, in serious cases, damage the overall structure of the combustor. Therefore, the research on the prevention and suppression of unstable combustion is of great significance to the normal operation of the engine [1,2,3,4,5]. As the most easily obtained unstable signal in engineering tests, the complex change of dynamic pressure (DP) is caused by the coupling of various physical processes in the combustor. Thus, many researchers have done a great deal of research on the mechanisms, characteristics, and early warnings of unstable combustion that are based on DP change characteristics [6,7,8,9,10,11].

The DP signal is the complete expression of the different characteristics of the combustor flow field. How to reverse the DP decomposition signal to show different flow field features is of great importance in studying the transformation and induction of the combustion state. Empirical mode decomposition (EMD) is a signal processing method that was proposed by Huang et al. [12]. Its main idea is to decompose the original signal into several intrinsic mode functions (IMFs). After multiple “filtering” steps, the original signal will be decomposed into multiple IMFs and a residue, and each order component will contain the local features of the original signal, which can extract the features of the original signal more accurately. The EMD signal decomposition method is different than fast Fourier transform (FFT) and discrete wavelet transform (DWT). The last two decomposition signals are based on frequency assignment [13], whereas EMD can be considered a decomposition that is based on certain internal features of the original signal [12]. EMD has been extensively used in various engineering fields such as mechanical failure diagnosis, seismic study, and ECG medical analysis. Zhao et al. [14] applied the method of combining multivariate EMD and full spectrum analysis to condition monitoring of rotating machinery, and found that this method can effectively diagnose the damage in the impeller and the rub of the rotor-stator. Singh et al. [15] applied EMD to obtain the multi-order IMF of the original signal from rotating machines. After analysis, the IMF is considered as having important fault information. Yang et al. [16] applied EMD to decompose the nonstationary signal of fault earthquakes, and after predicting and stacking several IMF signals, it was found that the error of short-term seismic prediction values under this method is small. Amoura et al. [17] examined the EMD decomposed logging noise signal IMF and reconstructed it, then determined the lithological characteristics of the formation. Boda et al. [18] applied EMD to filter the noise in the ECG signal, and the processed the signal that retained the original characteristics of ECG, and the denoising performance was higher than that of other methods. In combustion, a large number of academics have done a great deal of research on nonstationary signals using EMD. Yadav et al. [19] Used the method of combining EMD and hidden Markov model (HMM) to analyze the sound pressure signal of an internal combustion engine, they found that this method can realize the condition monitoring and fault diagnosis of the internal combustion engine after classifying the acoustic signals of the internal combustion engine with HMM when taking IMF as the feature vector. Lu et al. [20] used the IMF that was obtained by EMD decomposition of the pressure signal in the homogeneous charge compression ignition (HCCI) combustor to construct the feature vector to identify the feature parameters in the change of normal combustion and detonation combustion state. EMD has proven to be an effective way to extract functionality. Yao et al. [21] applied EMD to filter the low-frequency noise component and removed the influence of ambient noise on pressure sensor calibration. Bian et al. [22] calculated the matrix of high-frequency IMF and low-frequency IMF that were obtained by EMD decomposition of fuel spectral signal, and then combined that with UV spectrum to determine the content of carbohydrates in fuel samples. Yan et al. [23] applied EMD to decompose the impact flame noise signal of gasifier and found that the combustor system has the dynamic change characteristics from non-uniformity to uniformity. When unstable combustion occurs, the DP signal in the combustor changes complex, including the superposition of pressure pulsation that is caused by flow and combustion. Konle et al. [24] observed the interaction between the reaction and flow under different volumetric heat release rates by experiment and simulation and found that the volumetric expansion that was caused by heat release rate has an important impact on the convection field and flame stability. Xu et al. [25] studied the effect of swirl position on combustion instability by altering the swirl mixing distance, which will affect the frequency and growth rate of thermoacoustic instability. Steinberg et al. [26] studied the main flow structure in swirling combustion and found that the periodic shear pulsation and annular vortex shedding of flame structure are considerable reasons for unstable combustion.

Throughout this paper, we focus exclusively on the unstable combustion test of industrial central staged swirl combustor and gas fuel model swirl combustor. After decomposing the dynamic pressure signal in the combustor by EMD, the evolution characteristics of first order IMF components are compared. The other collected signals in different combustion states are analyzed by corresponding methods. Furthermore, the characteristics of each physical process are reconstructed in the complex change of DP signal. Finally, the feasibility of reverse evolution derivation is obtained by comparing the test results of two different combustors.

## 2. Experimental Facilities and Test Conditions

Unstable combustion includes two different states: combustion instability (CI) [27] and flame instability (FI) [28]. CI is caused by the positive feedback that is formed by the coupling of heat release rate and acoustic waves in the combustor, which will lead to an extremely high amplitude pressure pulsation, also known as oscillating combustion. In industrial combustors, it shows periodic pulsation of heat release rate and pressure with relatively high frequency compared to FI. FI is prone to produce in the lean fuel state of the combustor and it will be leading to deviate from the stable combustion state. This state will also occur when high-altitude aircraft adjusts the operating conditions, finally resulting in engine flameout; FI usually shows periodic shedding of flame at relatively low frequency [29]. In the bench test, the dominant frequency is concentrated at 10–40 Hz [30], at the same time, the pressure pulsation is obviously intermittent. FI is regularly the inducement of CI. If FI can’t lead to flameout, it may lead to CI. Through the above analysis, it can be considered that the change information of DP includes heat release rate pulsation, flame shedding, and flow field vortex pulsation when unstable combustion occurs. In order to study the change of connotation characteristics of the DP signal, two kinds of combustors, industrial central staged swirl combustor and gas fuel model swirl combustor, were tested.

### 2.1. Test Structure and Measurement System

**Figure 1:**
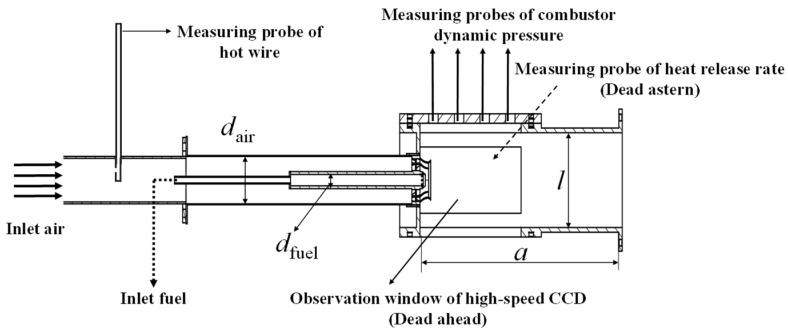
The structure and measurement layout of the M combustor.

The structure and layout of the gas fuel model swirl combustor are shown in Figure 1, which was divided into the intake section, combustor, and exhaust section. Air was supplied from the left to the right by external fans which enters the combustor through a two-stage axial cyclone through a pipe with an outer diameter of d_air_ = 50 mm. The average inlet velocity of the combustor was measured by a hot wire anemometer. The fuel was propane that was supplied by an external gas cylinder, which enters the combustor through 12 jet holes in the circumferential direction of the cyclone. The diameter d_fuel_ of the jet holes were 12 mm. The combustor was a cube with length a = 300 mm. Its cross-section shape was square, and the side length l = 100 mm. Quartz glass observation windows were set on both sides for shooting dynamic flame images and CH* light intensity signal acquisition. A total of four axially distributed dynamic pressure measuring probes were set at the top of the combustor to collect DP signals. The propane flow was regulated by AST10-H thermal gas mass flow controller. The accuracy of AST10-H is ±1.0% S.P (≥35% F.S) and ±0.35% F.S (<35% F.S), the repetition accuracy is ±1.0% F.S. The response time is 1 s. The combustor was recorded as M combustor.

**Figure 2:**
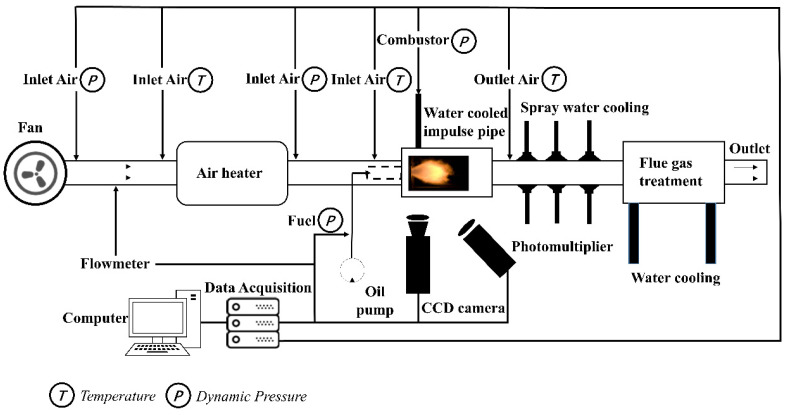
The structure and measurement layout of the L combustor.

**Figure 3:**
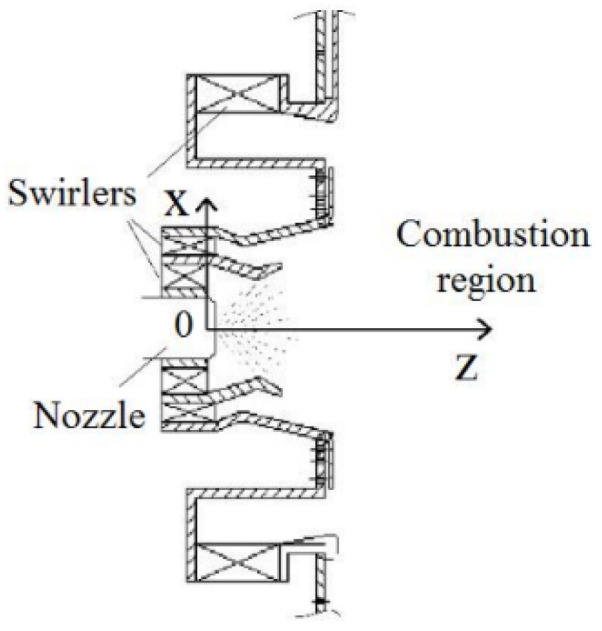
Structural diagram of swirl head.

The test system of industrial central graded swirl combustor is shown in Figure 2. The test was carried out in a normal temperature and pressure environment. The air was supplied by a Roots blower and heated by an electric heater before entering the combustor. The inlet flow was controlled by adjusting the fan frequency, and the differential pressure flowmeter was installed in front of the heater to measure the mass flow of air. The head of the combustor consisted of a primary radial stage cyclone and a two-stage axial duty stage cyclone; its structure is shown in Figure 3. The fuel was RP-3 aviation kerosene, which was fed through the fuel pump. The outlet gas of the combustor was cooled by spraying water and discharged into the atmosphere after flue gas treatment. The test measurements that were mainly collected were the signal of dynamic pressure, high-speed images, CH* intensity, and the temperature. Dynamic pressure measuring probes and temperature measuring probes were set in different parts of the whole combustor. In order to ensure the normal operation of the dynamic pressure sensors, a 350 mm long water-cooled pressure tube was used to measure the pressure pulsation of the combustor. An observation window was set in the burning zone to collect the CH* intensity signal and flame dynamic images. The pressure of the intake air and fuel were measured by pressure transmitters. The temperature of the intake air and combustor were measured by thermocouples. The type of thermocouples that was used was S-type. The combustor was recorded as L combustor.

In Figure 2, the circle T represents the position of the temperature measurement probe and the circle P represents the position of the dynamic pressure measurement probe.

The model of PCB113B28 dynamic pressure sensors were selected for the measurement, whose measurement range is 344.7 kPa and sensitivity is 14.5 mV/kPa. Meanwhile, their low frequency response is 0.5 Hz and non-linearity within 1.0% FS. The chemical fluorescence wavelength of CH* is 435.3 nm, so the CH* fluorescence signal was screened by a 435 nm ± 5 nm bandpass filter. The fluorescence intensity was measured by Hamamatsu photon CH348 photometric detector. The flame image was taken by an IDT-Y5 CCD high-speed camera. Its maximum frame rate is 1455 fps when the maximum resolution is 2336 × 1728, and the maximum achievable frame rate is 68,000 fps when the resolution is 2336 × 16. The data acquisition system adopted the NI CompactDAQ cDAQ-9178 high-speed data acquisition equipment, which supports 8 groups of acquisition modules for parallel acquisition. This paper selected multiple NI-9239 voltage acquisition modules to collect the above signals. The sampling rate of signals was 2 kHz. Each operating condition conducted multiple tests to eliminate test contingency.

### 2.2. Test Conditions

In order to analyze the DP pulsation characteristics under different combustion conditions in different combustors, the test conditions of M combustor and L are shown in Table 1 and Table 2, respectively.

M as a mechanism model combustor, mean that its air intake *Re* remained constant at 3802. *Re* is the Reynolds number which represents the ratio of inertial forces to viscous forces of a flow of liquid. The combustion mode is non-premixed. The equivalence ratio ϕ decreased gradually as propane decreases from MA01 to MA05. ϕ represents the ratio of the actual fuel–air ratio *f_a_* to chemically appropriate fuel–air ratio *f_s_*. The fuel–air ratio (*Far*) represents the ratio of fuel mass flow m_f_ to air mass flow m_a_. When ϕ > 1, it means a fuel-rich state; when ϕ < 1, it means lean-fuel state. The ϕ is the product of *Far* and theoretical air mass G_0_ that is required for complete combustion of 1 kg fuel. When the inlet air mass remains constant and the fuel type remains unchanged, subsequently the ϕ can be calculated through the change of fuel mass. In operating conditions of an M combustor, the ϕ changed from 0.185 to 0.074. Meanwhile, the computing formula of *Re*, ϕ, *Far* as following:

(1) Re=ρυL/μ

(2) $$\phi =\frac{f_{a}}{f_{s}}$$

(3) $$Far=\frac{m_{f}}{m_{a}}$$

ρ is the fluid density;
υ is the kinematic viscosity of a fluid;
L is the characteristic linear dimension;
μ is the dynamic viscosity of a fluid;

A total of three different intake air mass flow *W_a_* were set in the L combustor. The combustion mode is non-premixed. The intake air mass flow *W_a_* of each group remained constant at 100.2 g/s, 130.1 g/s, 140.0 g/s. There were five operating conditions that were contained in each group, where *Far* decreases as the group case number increases. The different operating conditions could change the different combustion states.

## 3. Introduction of Data Processing Method

### 3.1. Introduction to Empirical Mode Decomposition

The EMD decomposition idea first finds out the maximum and minimum points of the signal, then uses two cubic splines to fit the upper and lower envelope and obtains its average value. This step is repeated after subtracting the above average value from the original signal to obtain several IMF and a residue. When the residue is a function of the constant, monotonic slope, or unipolar value, the algorithm ceases. The decomposed IMF contains the information of each time characteristic scale. EMD decomposes the signal according to its time scale characteristics without preset primary function, which is different from Fourier decomposition and wavelet decomposition. It lends itself to analyze nonlinear and non-stationary signals, so it has outstanding advantages in processing unstable signals. The steps of the EMD algorithm are as follows:

(1) All local maximum points and all local minimum points in the original signal x(t) are obtained;
(2) The upper envelope $x_{\max}(t)$ and the lower envelope $x_{\min}(t)$ are obtained by fitting the extreme points with cubic spline interpolation, so that $x_{\min}(t)$ contents:
  (4) $$x_{\min}(t)\leq x(t)\leq x_{\max}(t),t\in \left[t_{1},t_{2}\right]$$
(3) The mean value is calculated for the upper envelope and the lower envelope:
  (5) $$m_{11}(t)=\frac{x_{\max}(t)+x_{\min}(t)}{2}$$
(4) Obtain signal local details $h_{11}(t)$:
  (6) $$h_{11}(t)=x(t)-m_{11}(t)$$
(5) If $h_{11}(t)$ contents the conditions are defined by IMF, take $h_{11}(t)$ as the first IMF $c_{1}(t)$, then $c_{1}(t)=h_{11}(t)$. If not, $h_{11}(t)$ is regarded as the original signal and the above steps are carried out until $h_{1m}(t)$ of the *m*-th time contents the conditions. Namely:
  (7) $$h_{1(m-1)}(t)-m_{1m}(t)=h_{1m}(t)$$
  where $m_{1m}(t)$ is the mean value of $h_{1(m-1)}(t)$ upper and lower envelope; $h_{1m}(t)$ contents the definition of IMF, that is the first IMF component screened, then:
  (8) $$c_{1}(t)=h_{1m}(t)=x(t)-\sum_{i=1}^{m}m_{1m}(t)$$
(6) The first IMF $h_{1m}(t)$ is eliminated from the original signal to obtain the residue $r_{1}(t)$, namely:
  (9) $$r_{1}(t)=x(t)-h_{1m}(t)$$
(7) Take $r_{1}(t)$ as the original signal and repeat the above steps to obtain multiple IMF and final residue $r_{n}(t)$:
  (10) $$\{\begin{array}{c} r_{2}(t)=r_{1}(t)-c_{2}(t)\\ r_{3}(t)=r_{2}(t)-c_{3}(t)\\ .\\ .\\ \begin{array}{c} .\\ r_{n}(t)=r_{n-1}(t)-c_{n}(t) \end{array} \end{array}$$
(8) When the decomposition process is completed, the original signal can be expressed as:
  (11) $$x(t)=r_{n}\left(t\right)+\sum_{i=1}^{n}c_{i}(t)$$

### 3.2. Introduction to Flame Image Boundary Identification Method

In order to analyze the boundary information in the flame image that is captured in the experiment, firstly, the median filter is selected to filter the noise signal in the image. The advantage of the median filter was that its nonlinear filtering technology for controlling noise is based on sorting statistical theory. Its basic idea is to replace the gray value of a pixel with the median value of its neighborhood pixel gray value, which doesn’t depend on those values that are vary widely from the typical value in the neighborhood. The expression of median filter function can be expressed as:

(12) g(x,y)=med{f(x−k,y−l),(k,l∈W)}

g(x,y) represents the processed image;

f(x,y) represents the original image;

W represents a two-dimensional template, it is usually 3 × 3, 5 × 5 areas, and can also be in different shapes, such as linear, circular, cross-shaped, etc. k and l represent the processing bits on the x-axis and y-axis, respectively.

**Figure 4:**
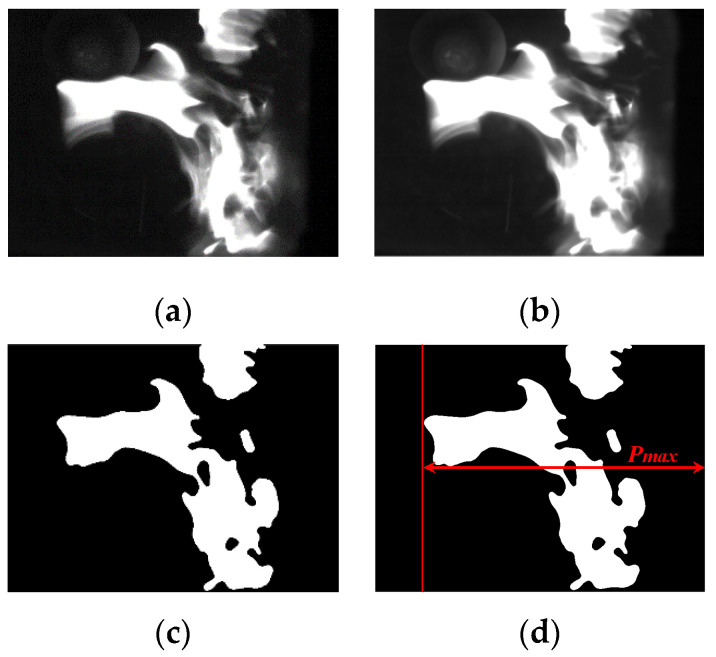
Analysis of the flame image: (**a**) The original image; (**b**) the image after median filter; (**c**) binarization of flame; and (**d**) the farthest pixel position of the flame.

The 5 × 5 was selected as the median value of the neighborhood that can filter the noise points and retain the flame edge details well. The processing result was changed from Figure 4a to Figure 4b.

For getting the farthest pixel of the flame, the image binarization that is based on threshold was used. Its basic idea is to replace all pixels with value of 1 (white) and 0 (black). If the pixel value of a point exceeds threshold, it converts to 1; else converts to 0. The threshold keeps at 0.3. The processing result was changed from Figure 4b,c. After the binary conversion of the flame image, the farthest pixel position of flame boundary can be sought out. The following Figure 4d shows the ultimate processing result of the farthest pixel position which is recorded as *P_max_*.

## 4. Analysis of Test Results

### 4.1. Analysis of Test Results of M Combustor

As a common diagnostic signal in engineering, the complex change of DP in combustor is caused by multiple physical processes. In this section, the DP signal will be analyzed in detail in the M combustor, and then other combustion diagnostic signals will be used to inverse the information of physical processes that are contained in the DP signal.

#### 4.1.1. Analysis of Dynamic Pressure Signal under Combustion

**Figure 5:**
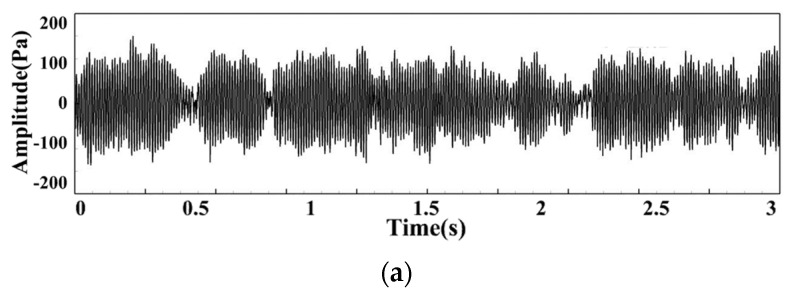

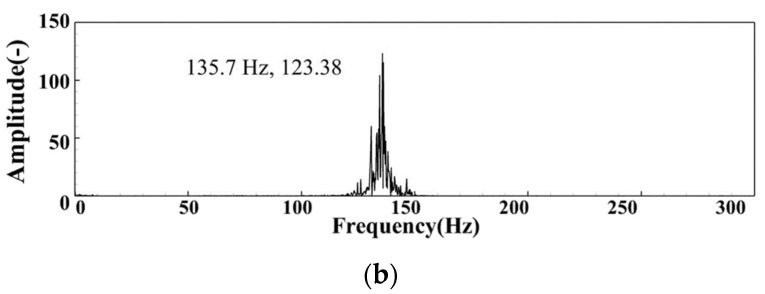
DP signal and spectrum within 2 s under MA02 operating condition: (**a**) DP signal of MA02; (**b**) Spectrum of DP signal.

Taking the DP signal under MA02 operating conditions as an example, Figure 5a shows the change of the DP in the combustor within two seconds under MA02 operating conditions. There are alternating high and low peaks within 3 s. Figure 5b shows the FFT spectrum of the DP signal under this operating condition, and the main frequency of the overall pulsation in the combustor is 135.7 Hz. The complex change of the original signal is caused by multiple excitation sources. Its change contains more information, and the detailed information cannot be obtained from the FFT spectrum alone. The DP signal is decomposed by EMD to obtain the characteristic information of the internal excitation sources.

**Figure 6:**
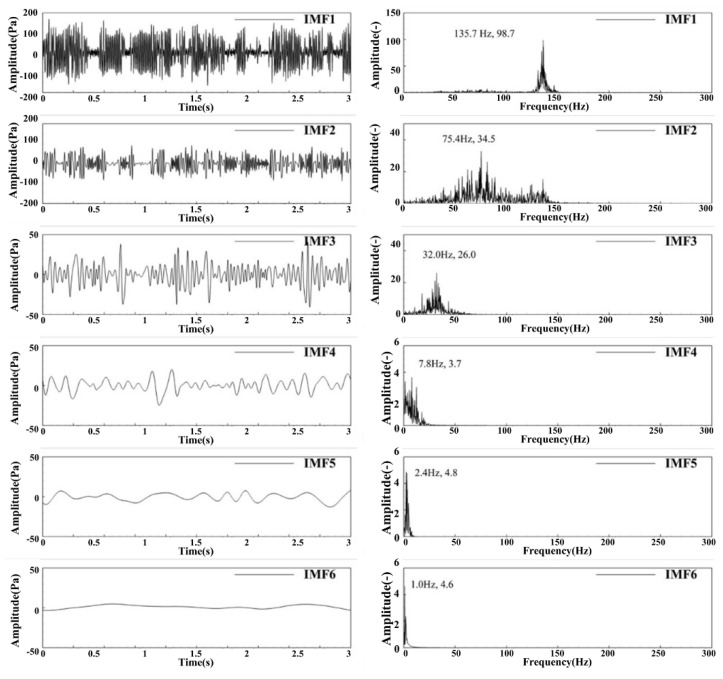
First six order IMF signals and the spectrum of the DP signal under MA02 operating condition.

Figure 6 shows each order of IMF after EMD decomposition of the DP signal and the FFT spectrum of the first six orders of IMF. The pulsation energy is concentrated in the first three order IMF, in which IMF1 is approximately consistent with the amplitude and waveform of the original signal, indicating that IMF1 energy is dominant in DP pulsation. The pulsation peaks of IMF2 and IMF3 are close, and the frequency of IMF3 is significantly lower than that of IMF2. The pulsation of IMF4-IMF6 is small and the main frequency is within 10 Hz. There is an obvious pulsating dominant frequency in IMF1-3, the dominant frequency gradually decreases, and the amplitude of the dominant frequency also gradually decreases. There is no obvious pulsating dominant frequency in IMF4-6, which will not be considered.

**Figure 7:**
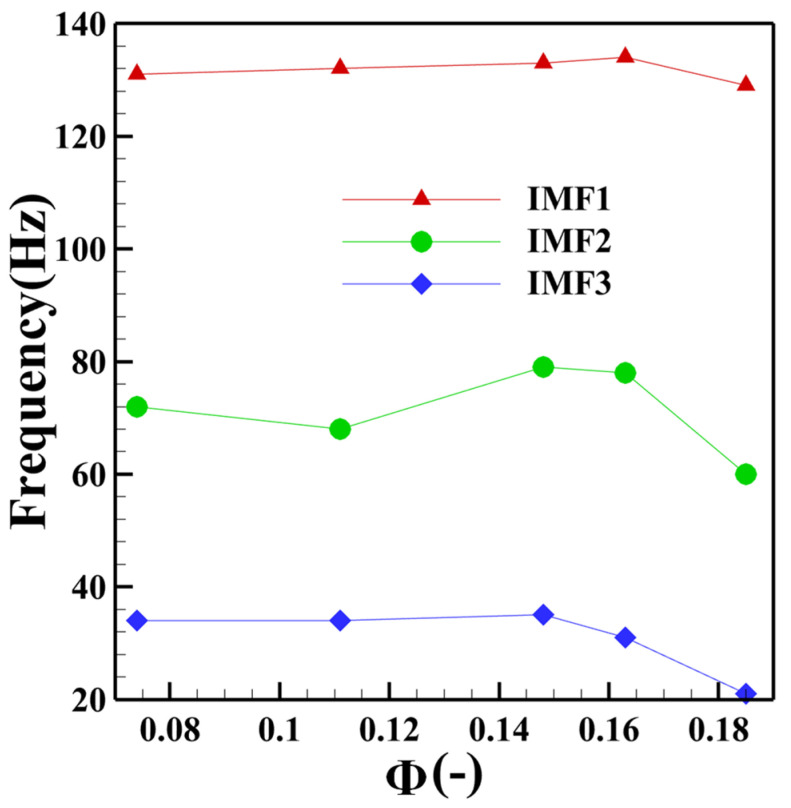
Change of the main frequency of IMF1-3 under operating condition of the MA group.

Figure 7 shows the variation law of IMF1-3 dominant frequency with equivalence ratio under MA group operating conditions. The main frequency of IMF1 pulsation gradually decreases with the decrease of the equivalence ratio, and finally remains stable near 130 Hz. The main frequency of IMF2 pulsation fluctuates up and down in the range of 60–80 Hz. With the decrease of the equivalence ratio, the pulsating dominant frequency of IMF3 gradually increases and remains stable near 34 Hz.

In order to analyze the characteristics of the internal excitation source of DP pulsation, it is necessary to analyze the characteristics of other signal pulsation in the combustor. In the following, the macro flow field pulsation under internal cold conditions, CH* signal, and the flame image signal during combustion will be analyzed.

#### 4.1.2. Analysis of Dynamic Pressure Signal under Cold State

**Figure 8:**
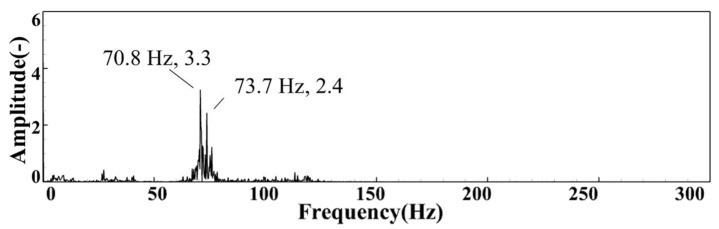
The spectrum of DP pulsation in the combustor under cold conditions.

Figure 8 is the FFT spectrum of the DP signal in the combustor under the cold conditions corresponding to intake *Re* = 3802. The main reason for the pulsation in the cold macro flow field is that the mainstream air passes through the blades and forms a precession vortex after being sheared. Finally, it results in an uneven overall flow field and further forms continuous pulsation. Under this cold condition, the main frequency of pulsation is concentrated in 70.8–73.7 Hz.

#### 4.1.3. Analysis of CH*

**Figure 9:**
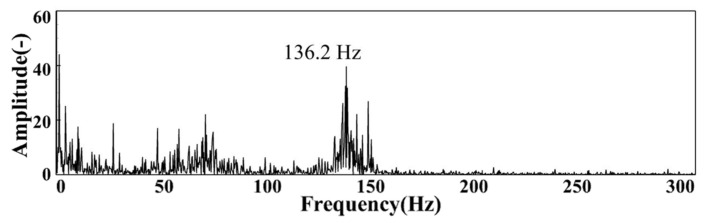
The spectrum of CH* pulsation under MA02 operating conditions.

**Figure 10:**
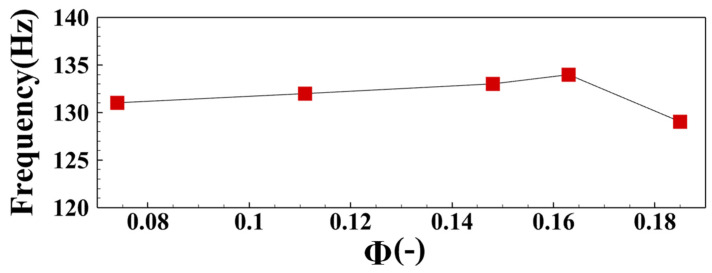
Variation results of CH* pulsation dominant frequency with the equivalence ratio.

Figure 9 is the FFT spectrum of the CH* signal under MA02 operating conditions. In this operating condition, the pulsating energy is mainly concentrated in the broadband near 136.2 Hz. Figure 10 shows the variation of the CH* pulsation dominant frequency with the equivalence ratio under operating conditions of group MA. It can be seen that when the intake *Re* is kept constant and the equivalence ratio is gradually reduced, the main frequency of CH* pulsation will first increase and then gradually decrease, which is consistent with the change of the main frequency of IMF1 pulsation.

#### 4.1.4. Analysis of Flame Images

**Figure 11:**
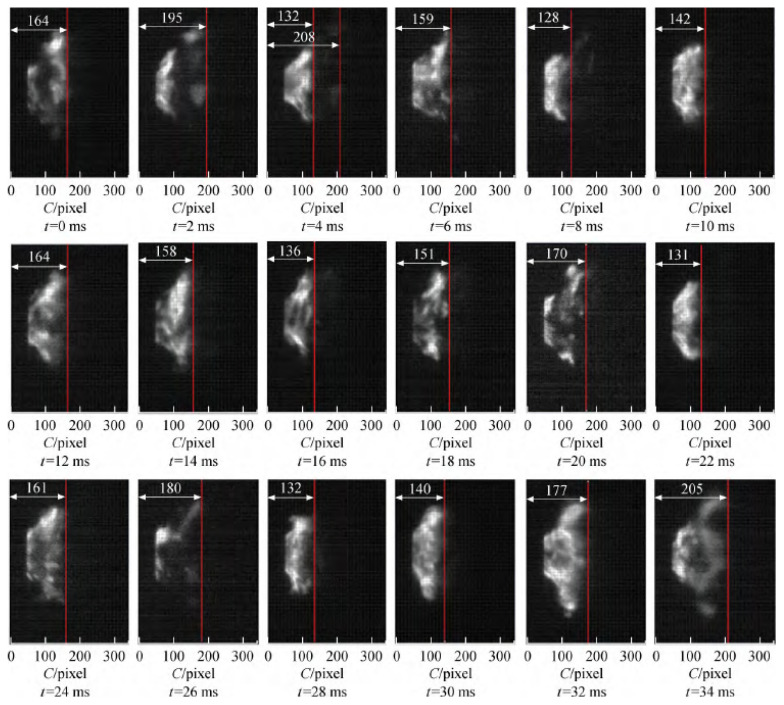
Flame image changes within 0–34 ms of the M combustor.

Figure 11 is the flame change image within 0–34 ms under MA2 operating conditions. The change of the flame surface is complex. While shaking at high frequency, part of the tail flame leaves the main body and floats downstream. From the flame image results, there is a dark area between the flame tail and the main flame at the outlet of the cyclone when the flame falls off, and the flame pixel position is high at this time. In Figure 11, there is an obvious flame falling off process in *t* = 2 ms and *t* = 4 ms. The main flame is located at 132 pixels, and the farthest position when the flame falls off is 208 pixels. When there is high-frequency jitter on the flame surface, the position of the flame surface will also change, but the farthest position of the change will not exceed 180 pixels. Based on the above analysis, it can be seen that the flame also falls off when *t* = 34 ms. It can be estimated that the flame shedding frequency is about 33 Hz.

**Figure 12:**
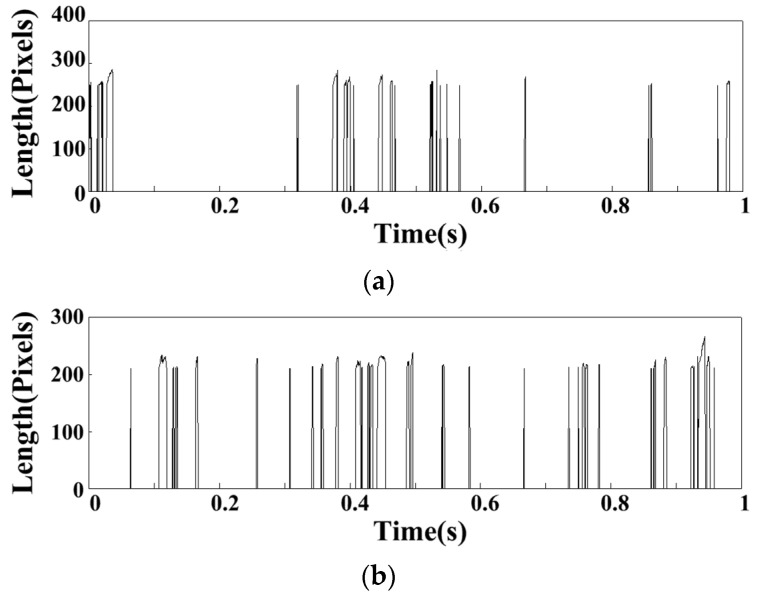

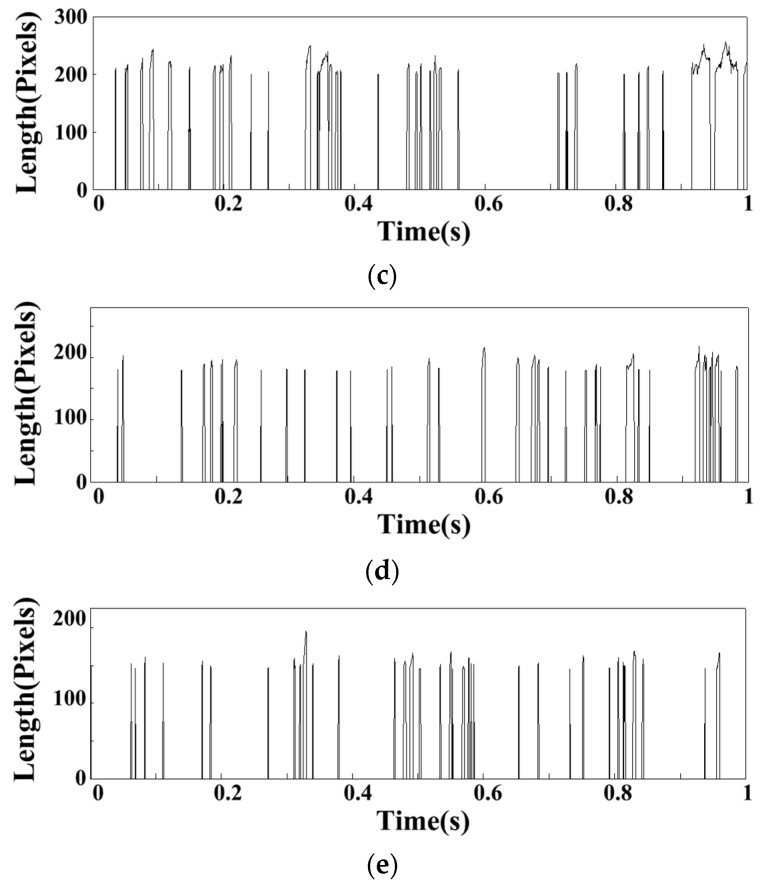
Statistics of flame shedding under operating conditions of group MA: (**a**) MA01; (**b**) MA02; (**c**) MA03; (**d**) MA04; (**e**) MA05.

Figure 12 shows the statistical results of the flame shedding signal in all the operating conditions of group MA. In the MA01 operating conditions, the farthest position of the flame is 300 pixels. With the decrease of fuel–air ratio, the farthest position of the flame will gradually decrease. In MA05 operating conditions, the farthest position of the flame is 200 pixels. It indicates that the flame length is longer under the condition of high equivalence ratio. According to the statistics, the flame shedding frequencies within 1 s under five operating conditions are 21 Hz, 31 Hz, 35 Hz, 33 Hz, and 33 Hz.

Through the above analysis, it was found that the change of IMF1 pulsation dominant frequency corresponding to CH* signal pulsation dominant frequency is approximately the same, indicating that IMF1 may have the pulsation characteristics of heat release rate. The main frequency of IMF2 pulsation is close to that of DP pulsation under cold conditions, indicating that IMF2 may characterize the pulsation The main frequency of IMF3 pulsation is consistent with the flame shedding frequency, indicating that IMF3 may characterize the flame shedding frequency. In order to verify this conclusion, the combustion characteristics in the industrial central staged swirl combustor are verified in the next section.

### 4.2. Analysis of Test Results of L Combustor

Based on the analysis of the DP signal and other multiple characteristic signals in the M combustor, it was found that the IMF components of each order of the DP signal after EMD decomposition may correspond to other physical characteristics of the combustor, respectively. In order to verify this thesis, this section will perform the same processing on the DP signal, CH* signal, and high-speed flame images of the L combustor.

#### 4.2.1. Analysis of Dynamic Pressure Signal under Combustion

**Figure 13:**
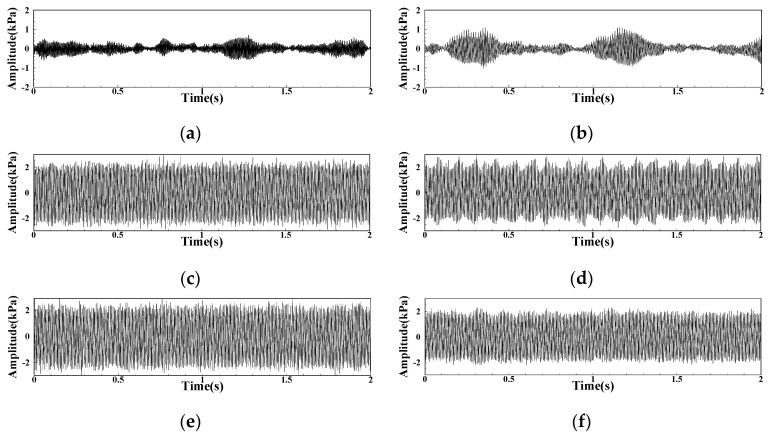
DP signal within 0–2 s under each operating condition of group LA: (**a**) LA01; (**b**) LA02; (**c**) LA03; (**d**) LA04; (**e**) LA05; (**f**) LA06.

Taking the LA group as an example for the DP signal of the L combustor, Figure 13 shows the change result of the DP signal under the LA01-LA06 operating conditions. Under the operating conditions of LA01 and LA02, the fuel–air ratio is high, and the pressure pulsation waveform has obvious intermittence. Its waveform is the DP waveform signal under the classical FI state. When the fuel–air ratio is further reduced to the LA03 operating condition, the overall amplitude of the DP signal increases sharply and maintains in CI state. The signal changes of LB and LC groups are similar.

**Figure 14:**
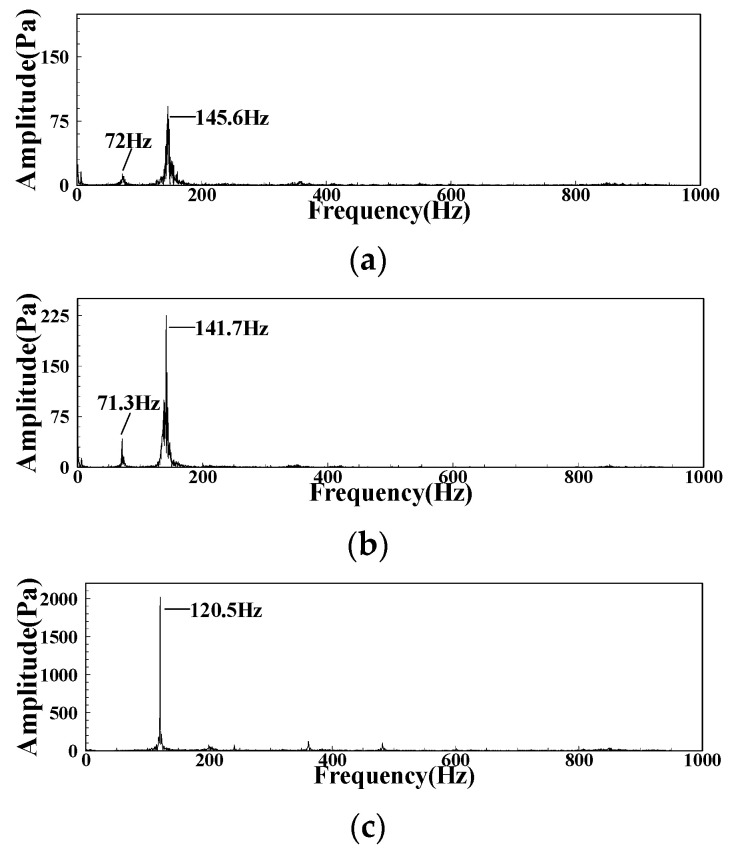
The spectrum of the DP signal under different operating conditions: (**a**) LA01; (**b**) LA02; (**c**) LA03.

The spectrum result of DP signal in LA01-LA03 is obtained after FFT calculation, as shown in Figure 14. In the LA01 and LA02 operating conditions, the pulsation peak will increase with the decrease of the fuel–air ratio, and the frequency of the main peak will decrease, but all around 140 Hz. The sub peak near 70 Hz also has similar changes. In LA03, due to the influence of CI, the pulsation peak will increase sharply. Simultaneously, there is a single main peak of 120 Hz in the pulsation spectrum and no other obvious secondary peak. The other operating conditions of group LA are in the CI state, and the pulsation spectrum is similar to LA03.

**Figure 15:**
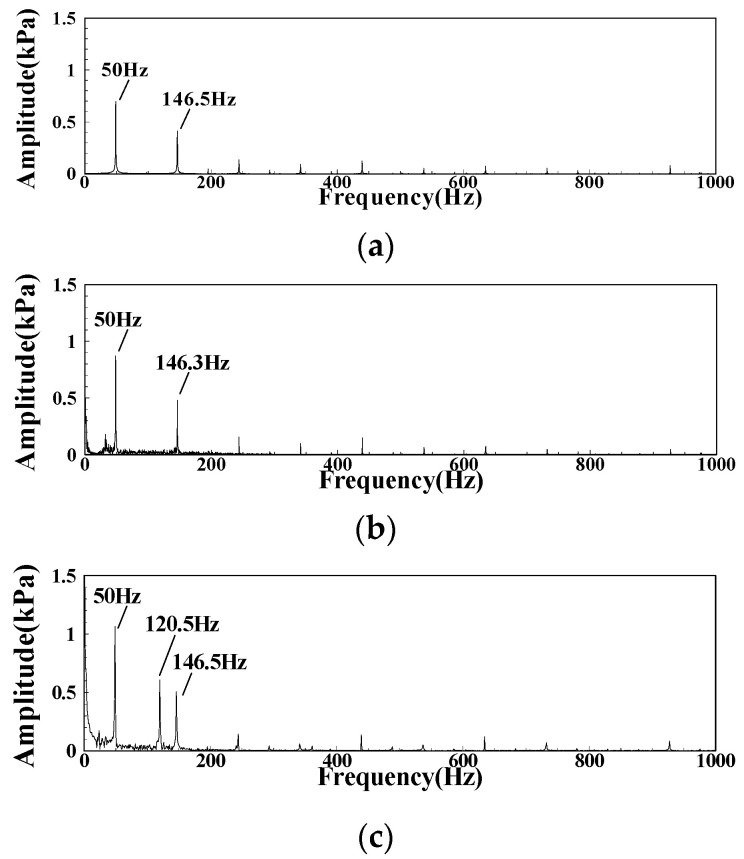
The spectrum of fuel pressure signals under different operating conditions: (**a**) LA01; (**b**) LA02; (**c**) LA03.

In the CI state, the main frequency of the DP pulsation is around 120 Hz, which is the eigenfrequency that was determined by the structure of the combustor itself. In the FI state, the main frequency of the DP pulsation is 140 Hz. In order to consider the cause of this frequency, the frequency spectrum of the inlet fuel pressure pulsation signal in LA01-LA03 is calculated, and the results are shown in Figure 15. There are main peaks and sub peaks of 50 Hz and 146 Hz in the FI state. The 50 Hz main peak is consistent with the power frequency, and 146 Hz is the pulsating main frequency when the fuel pump is supplying fuel. With the decrease of the fuel–air ratio, when the FI state changes to the CI state, 120 Hz acoustic oscillation frequency will appear under the influence of oscillation combustion, and its pulsation intensity will be higher than the influence of the fuel pump on fuel supply pulsation.

**Figure 16:**
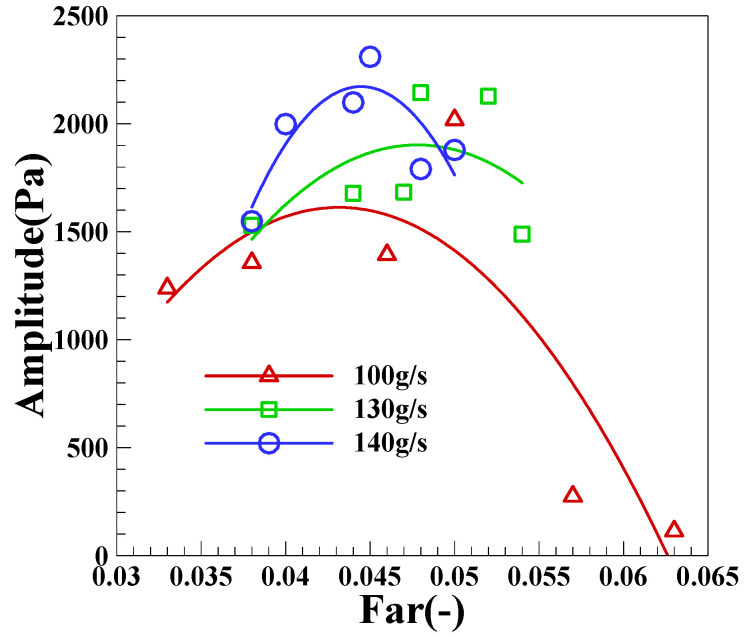
Variation of the DP peak value under different inlet air mass flow.

Figure 16 shows the variation results of the DP pulsation peak value under different inlet air mass flow. When *W_a_* of group LA is 100 g/s, the peak value under a high fuel–air ratio maintaining in FI state will be far less than the pulsation peak value under CI state. When the fuel–air ratio is reduced to CI state, the DP peak value will increase sharply and the oscillation intensity of combustor will increase greatly; Among the three groups of intake air, when the combustion is in CI state, there will be a critical fuel–air ratio to maximize the DP peak. When the fuel–air ratio continues to decrease on the basis of this fuel–air ratio, the DP peak will gradually decrease. The decrease of the DP peak value is mainly because when the fuel–air ratio decreases to a certain extent, the energy density in the combustor will decrease with the decrease of fuel–air ratio, which further leads to the decrease of oscillation intensity.

**Figure 17:**
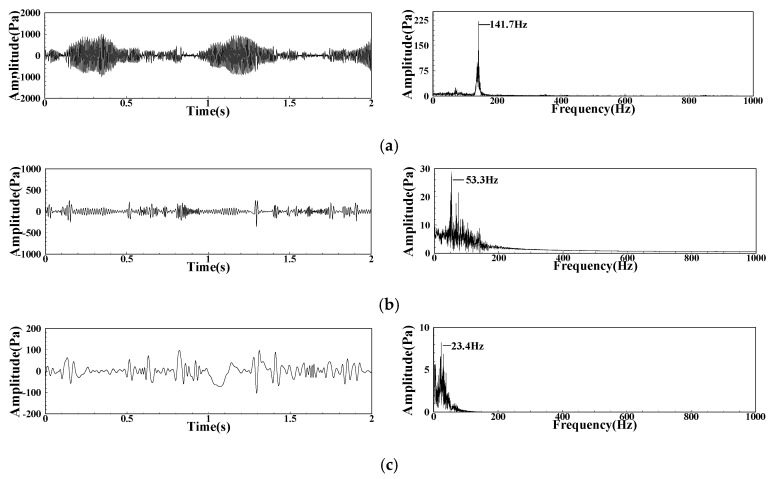
Waveform and spectrum of each component of DP signal under LA02: (**a**) LA02 IMF1; (**b**) LA02 IMF2; (**c**) LA02 IMF3.

LA02 is in the middle of the transition from FI to CI. The intermittent waveform signal is the common influence result of multiple complex factors in the combustor. The original signal contains rich information of multiple physical processes in the combustor. After EMD decomposition of the DP signals, a total of seven order components can be obtained. The energy is mainly concentrated in the first three orders, and the other low order amplitudes and dominant frequencies are too small to be considered. The waveform and spectrum of the first three order components are shown in Figure 17. The IMF1 waveform is similar to the original pressure fluctuation waveform and amplitude. The main frequency of the two pulsations is the same, which retains the main characteristics of the original signal, but there is no sub peak of 71.3 Hz. The IMF2 waveform still has strong intermittence, and its pulsation energy is concentrated in a wide frequency band near 53 Hz. The main frequency of the IMF3 pulsation is concentrated in the low frequency band near 23 Hz. Generally, it can be considered that the three components that are obtained after EMD decomposition are relatively independent, corresponding to three independent physical processes.

**Figure 18:**
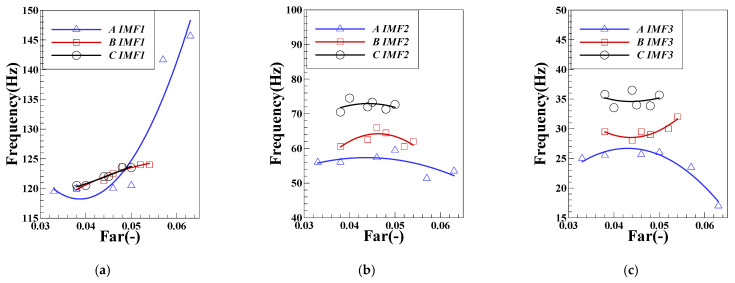
Variation of the pulsation dominant frequency of the DP components:(**a**) IMF1; (**b**) IMF2; (**c**) IMF3.

**Figure 19:**
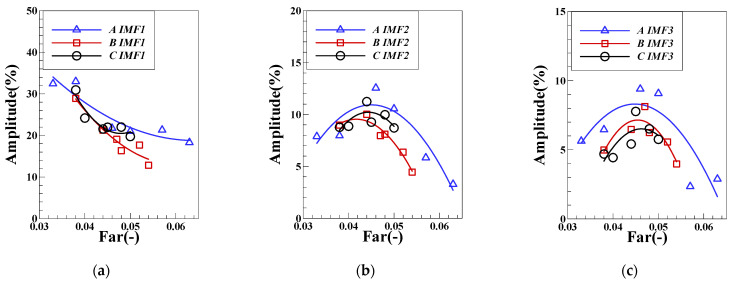
Variation of the proportion of pulsation peak energy of each order component of DP: (**a**) IMF1; (**b**)IMF2; (**c**) IMF3.

According to the above treatment method, after statistics of other operating conditions in the test, the changes of each order component of pressure pulsation under different mass flow are obtained, as shown in Figure 18. The pulsating dominant frequency of IMF1 changes greatly under the air intake of group LA, when the fuel–air ratio is high which maintaining in the FI state and the pulsating dominant frequency is higher than that in the CI state with low fuel–air ratio. In the CI state, the inlet air mass flow has little influence on IMF1, and its variation range of the main frequency is concentrated near the eigenfrequency of longitudinal acoustic in 120 Hz. When the inlet air mass flow remains unchanged, the pulsating dominant frequency of IMF2 changes slightly around a fixed base value. With the increase of the intake air flow, the base value of each operating condition will gradually increase. When changing state from FI to CI in the operating conditions of group LA, the main frequency of IMF3 pulsation will gradually increase from 17 Hz to 23 Hz until it maintains at 26 Hz. Through the above analysis, the first three order IMF pulsation main frequencies of pressure pulsation under FI and CI states will have obvious variation characteristics. When the main frequency of IMF1 is in lean fuel–air ratio maintaining in the CI state, it tends to the natural frequency of the system under each air intake. The dominant frequencies of IMF2 and IMF3 have an obvious positive relationship with the mass flow of intake air/*Re*, indicating that the variation characteristics of the physical process corresponding to these two components are positively correlated with the average velocity of the flow field. In the subsequent part, the spectrum characteristics of the component signal are mapped to the appropriate physical processes. Figure 19 shows variation of the proportion of the pulsation peak energy of each order component of DP. The proportion of IMF1 is the highest among the first three order components and will gradually increase with the gradual decrease of the fuel–air ratio. The results show that the peak value of IMF1 is positively correlated with the degree of unstable combustion. With the transition of combustion state from FI to CI, the proportion of physical process that are represented by IMF1 gradually increases. The change law of the main frequency energy proportion of IMF2 and IMF3 is similar. With the decrease of the fuel–air ratio, there will be a trend of first increasing and then decreasing, and there will be a peak at a certain critical fuel–air ratio. The results of other characteristic signals in the L combustor will be analyzed in detail.

#### 4.2.2. Analysis of Dynamic Pressure Signal under Cold State

**Figure 20:**
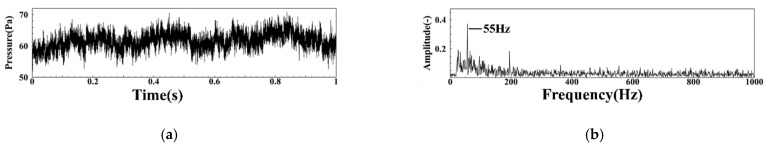
DP spectrum in combustor without inlet air: (**a**) DP signal; (**b**) Spectrum.

The macroscopic fluctuation that is caused by the flow field structure will significantly affect the change of pressure fluctuation. In order to analyze the influence of the flow field structure on DP in the L combustor, the DP signal in the cold state without ignition is analyzed. In case of no ventilation, the DP signal in the combustor within 1 s is shown in Figure 20a. At this time, due to the influence of background environmental noise, the low-frequency pulsation part of the DP signal will be doped with high-frequency pulsation. In order to reduce the influence in the background noise, the dimensionless spectrum of the original pulsating signal is calculated by FFT after filtering. The results are shown in Figure 20b. The overall DP spectrum distribution is close to the white noise distribution, but there is a pulsating main frequency near 50 Hz in the low-frequency part, which may be related to the inherent characteristics of the combustor.

**Figure 21:**
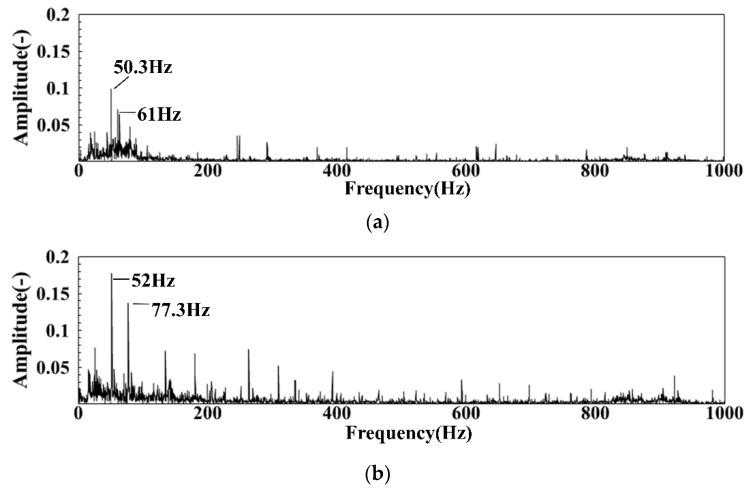
The DP spectrum of cold state under different air intakes: (**a**) The spectrum of cold DP in group LB; (**b**) The spectrum of cold DP in group LC.

The dimensionless DP pulsation spectrum of the intake air flow of groups LB and LC is obtained after a similar noise reduction treatment for the DP signal in the case of only the air supply without ignition as shown in Figure 21a,b. There is a pulsating dominant frequency near 50 Hz in the frequency spectrum under the two air intakes, which is consistent with the pulsating dominant frequency when there is no ventilation, indicating that 50 Hz is a fixed power frequency. There is a sub-peak dominant frequency of 61 Hz and 77.3 Hz in the DP spectrum of each air intake. The reason for this result is that when the inlet air passes through the cyclone blade, it will produce stress change after being subjected to cyclone shear, which will further cause the change of the DP pulsation dominant frequency.

**Figure 22:**
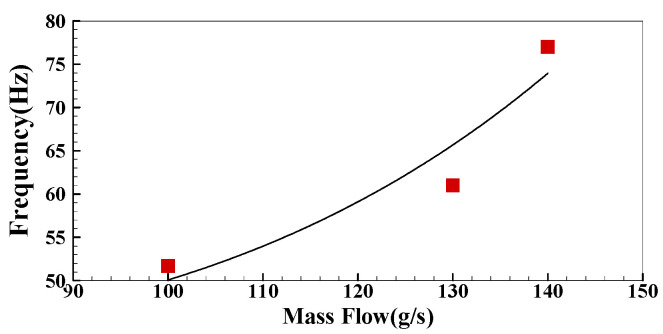
Variation of the secondary dominant frequency of cold DP under different inlet flows.

According to the above method of noise reduction, the change results of the pulsation dominant frequency that are caused by flow field structure in other operating conditions are shown in Figure 22 below. It can be seen that with the increase of inlet air mass flow, the dominant frequency of DP pulsation will gradually increase in the cold state. At the same time, the frequency variation increases nonlinearly with the variation of the inlet air mass flow. The change of the dominant frequency under different inlet air flows is approximately consistent with IMF2 above, indicating that IMF2 contains the macroscopic pulsation information of cold flow field structure.

#### 4.2.3. Analysis of CH* Signal

The most important source of pressure pulsation in the combustion flow field is the instantaneous pressure change of a premixed gas micro cluster during a violent chemical reaction (combustion), which is a monopole source term. There are a large number of micro clusters with a high heat release rate in the whole combustion zone to form a composite pressure pulsation source term. In this paper, CH* filter and photometric detector are used to collect the pulsation information of the heat release rate. However, this CH* signal is the overall pulsation characteristic that is formed by the movement of the heat release rate micro clusters. Theoretically, the CH* signal pulsation is the comprehensive effect of the gas micro cluster heat release rate pulsation and the combustion space flow field structure pulsation that are based on Lagrange’s point of view.

**Figure 23:**
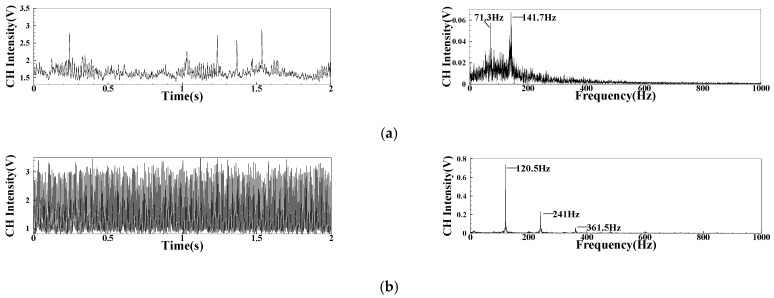
The CH* signal and spectrum of different combustion conditions: (**a**) The spectrum of CH*pulsation under the FI state; (**b**) The spectrum of CH* pulsation under the CI state.

In the test, the pulsation signal of CH* intensity and its spectrum under the FI state and CI state are shown in Figure 23. The CH* signal varies greatly under different states. Under the condition of a high fuel–air ratio, the overall intensity is concentrated between 0–2 V with intermittent short time scale. The intensity of the CI state will not appear with intermittent characteristics such as the FI state, and the overall amplitude is concentrated at 0–3 V. From the spectrum results, the intensity of the CH* pulsation is small in the FI state, and there will be no obvious single peak dominant frequency. In the CI state, there exists a single peak dominant frequency in the spectrum of CH* signal, and the pulsation peak will be much larger than that of FI.

**Figure 24:**
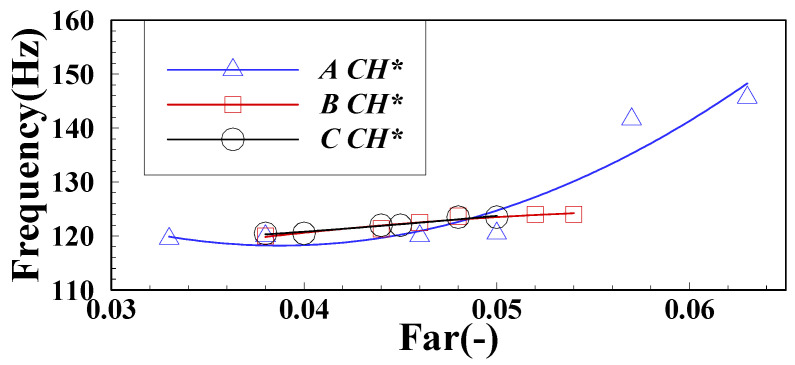
Variation of CH* pulsation frequency.

Through the analysis of the IMF1 and CH* signals in the FI state, it is found that the frequency changes are consistent. The changes of the CH* pulsation dominant frequency under three different intake mass flows are obtained after statistics of the CH* pulsation dominant frequency under all operating conditions, as shown in Figure 24. From the results, the dominant frequency of the CH* pulsation in the FI state with high fuel–air ratio will gradually decrease with the decrease of fuel–air ratio and maintain near 120 Hz in the CI state. After comparing the change of the pulsating dominant frequency of CH* with that of IMF1 of DP in Figure 18a above, it is found that the frequency change of the two signals is completely consistent. It is further deduced that the IMF1 pulsation component of DP can represent the change of pulsation characteristics of the heat release rate.

#### 4.2.4. Analysis of Flame Images

**Figure 25:**
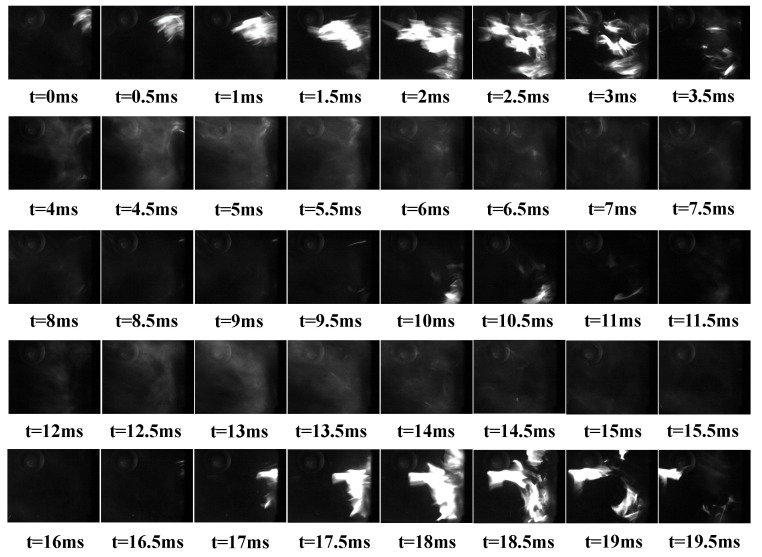
Flame change images of the L combustor.

A major factor in the complex change of DP signals is flame shedding. The flame will move downstream with the air flow during combustion, and the flame will be gradually stretched. When this phenomenon lasts for a certain time, part of the flame will separate from the main body of the flame to form flame shedding. This process will change the position of high temperature mass in the combustor, resulting in uneven space temperature. This section analyzes the characteristics of flame shedding in the L combustor. The following Figure 25 shows that 40 of the 150 flame change images that were selected from the results have flame off, and the phenomenon of flame falling off occurs about twice (t = 2 ms–3 ms and t = 18 ms–19 ms). In the reaction zone of the combustor, there is an obvious periodic alternation of light and shade. The reason for this phenomenon is that when the flame luminous high-temperature zone is affected by the swirling flow, it will flow along the axial direction and move in the circumferential direction at the same time, further forming the uneven circumferential distribution. In addition, there are unburned gas masses in the reflux zone that will be ignited, the above reciprocating motion of the flame leads to three types of flame images in the shooting results: bright flame, no bright flame exists a high-temperature mass, a dark area. Part of the bright flame will leave the flame body at the root of the cyclone and extend to the area outside the center. There are many influencing factors leading to flame shedding, including the gradual stretching of the flame surface that is caused by the shear stress of the mainstream air under the action of swirling flow, the extrusion deformation of the flame that is caused by pressure pulsation, and the gradient change in the reflux area. The image shooting frequency is 2000 Hz, and the frequency is about 2 × 2000/150~26.7 Hz.

**Figure 26:**
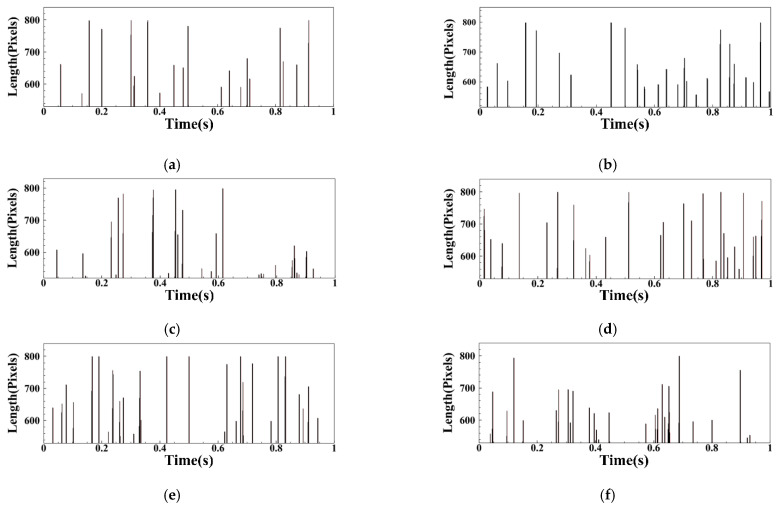
Statistics of flame shedding under operating conditions of group LA: (**a**) LA01; (**b**) LA02; (**c**) LA03; (**d**) LA04; (**e**) LA05; (**f**) LA06.

From the above analysis, when the flame sheds, there will be obvious flame clusters shedding. The pixels that are farthest from the cyclone in the statistical image are used as the basis for judging the flame shedding. After the above analysis and screening of the flame images under all operating conditions in the LA group, the flame shedding length within 1 s is shown in Figure 26. It can be seen that the flame shedding interval is irregular, and the flame length has an approximate sine wave change when the flame shedding occurs. The frequency of flame shedding in LA01-LA06 is 20, 24, 25, 26, 27, and 26 Hz after statistics. It can be seen that with the FI state developing into the CI state, the flame shedding frequency will gradually increase and basically remain 26 Hz in CI state.

## 5. Conclusions

In this paper, the DP signal, heat release rate, and the flame high-speed images in a gas fuel model combustor and an industrial central staged swirl combustor are studied. In the operating conditions that were studied in this paper, the following conclusions can be obtained:

(1) There are two kinds combustion states in an industrial combustor (flame instability and combustion instability). The diversities of the amplitude and waveform of dynamic pressure are obviously different in the two combustion states. The intermittence decreases gradually with the state changing from flame instability to combustion instability.
(2) The characteristics of the dynamic pressure pulsation under cold state mainly is influenced by swirler, which increases with the increases of the mass flow of intake air.
(3) In two kinds of non-premixed swirl combustors, it is found that the pulsating energy of the dynamic pressure concentrated in the first three order IMF after EMD decomposition. The main frequency of heat release rate pulsation, pressure pulsation characteristics under cold state, and flame shedding correspond to the main frequency of IMF1-3 pulsation, respectively. The frequency of flame shedding is below 40 Hz [30]. By contrasting the peak value of IMF1-3 and the proportion of pulsation peak energy of each order component of DP, the most important factor affecting the pressure pulsation is the contribution of the pulsation of heat release rate and the influence of flame flameout and the flow field pulsation is small.

## Figures and Tables

**Table 1:** Test conditions of the M combustor.

Case	*Re*/(-)	*Φ*
MA01	3802	0.185
MA02	3802	0.163
MA03	3802	0.148
MA04	3802	0.111
MA05	3802	0.074

**Table 2:** Test conditions of the L combustor.

Case	*W_a_*/(g/s)	*Far*
LA01	100.2	0.063
LA02	100.2	0.057
LA03	100.2	0.050
LA04	100.2	0.046
LA05	100.2	0.038
LA06	100.2	0.033
LB01	130.1	0.054
LB02	130.1	0.052
LB03	130.1	0.048
LB04	130.1	0.046
LB05	130.1	0.044
LB06	130.1	0.038
LC01	140.0	0.050
LC02	140.0	0.048
LC03	140.0	0.045
LC04	140.0	0.044
LC05	140.0	0.040
LC06	140.0	0.038

## Data Availability

Not applicable.
